# Socio-economic determinants of healthcare costs in early life: a register-based study in the Netherlands

**DOI:** 10.1186/s12939-021-01589-x

**Published:** 2022-01-12

**Authors:** Marije van der Hulst, Suzanne Polinder, Rianne Kok, Peter Prinzie, Marijke W. de Groot, Alex Burdorf, Loes C. M. Bertens

**Affiliations:** 1 Department of Obstetrics and Gynecology, Erasmus MC, University Medical Center Rotterdam, P.O. Box 2040, 3000 CA Rotterdam, the Netherlands; 2 Research Group Transforming Youth Care, The Hague University of Applied Sciences, The Hague, The Netherlands; 3 Department of Public Health, Erasmus MC, University Medical Center Rotterdam, Rotterdam, the Netherlands; 4 Department of Psychology, Education and Child Studies, Erasmus University, Rotterdam, the Netherlands; 5 Department of Obstetrics and Gynecology, Elisabeth Twee Steden Hospital, Tilburg, the Netherlands

**Keywords:** Socioeconomic aspects of health, Child health, Healthcare costs, Preterm birth, Small for gestational age

## Abstract

**Background:**

Children with low socioeconomic status (SES) have an increased risk of a suboptimal start in life with ensuing higher healthcare costs. This study aims to investigate the effects of individual- (monthly household income) and contextual-level SES (household income and neighborhood deprivation), and perinatal morbidity (preterm birth and small for gestational age ((<10th percentile), SGA)) on healthcare costs in early life (0–3 years of age).

**Methods:**

Individual-linked data from three national registries (Perinatal Registry Netherlands, Statistics Netherlands, and Healthcare Vektis) were obtained of all children born between 2011 and 2014 (*N* = 480,471) in the Netherlands. Binomial logistic regression was used to model annual healthcare costs as a function of their household income (per €1000), neighborhood deprivation index (range − 13.26 – 10.70), their perinatal morbidity and demographic characteristics. Annual healthcare cost were dichotomized into low healthcare costs (Q1-Q3 below €1000) and high healthcare costs (Q4 €1000 or higher).

**Results:**

Children had a median of €295 annual healthcare costs, ranging from €72 to €4299 (5–95%). Binomial logistic regression revealed that for every €1000 decrease in monthly household income, the OR for having high healthcare costs is 0.99 (0.99–0.99). Furthermore, for every one-unit increase in neighborhood deprivation the OR for having high healthcare costs increase 1.02 (1.01–1.02). Finally, the model revealed an OR of 2.55 (2.48–2.61) for preterm born children, and an OR of 1.44 (1.41–1.48) for children SGA, to have high healthcare costs compared to their healthy peers.

**Conclusion:**

More neighborhood deprivation was directly related to higher healthcare costs in young children. On top of this, lower household income was consistently and independently related to higher healthcare costs. By optimizing conditions for low SES populations, the impact of low SES circumstances on their healthcare costs can be positively influenced. Additionally, policies that influence more timely and appropriate healthcare use in low SES populations can reduce healthcare costs further.

## Introduction

The Developmental Origins of Health and Disease (DOHaD) paradigm describes how adverse exposures during pregnancy can have long-lasting effects on the developing fetus [1–3]. Apart from genetic background and hereditary predispositions, socioeconomic status (SES) is critical to fetal and infant development [4, 5]. Children of low SES backgrounds are at increased risk for perinatal mortality and are more often born preterm or small for gestational age ((SGA); with a birthweight below the 10th percentile, adjusted for gestational age and fetal sex) [6, 7]. Furthermore, children born in families with low SES are more likely to report unhealthy behavior and suboptimal (mental) health in later life, creating an intergenerational transmission of health disparities [2, 3, 8–14]. Additionally, individuals of lower SES often cluster together in deprived neighborhoods, in which there is an accumulation of social and economic risk factors, and residential instability, negatively impacting the mental, physical, and overall health of its residents [10, 11, 15]. Moreover, insufficient family resources limit not only adequate nutrition and infant stimulation opportunities, but also reduce parental time for adequate childcare and nurturance, obstructing all essential requirements for healthy child development [4]. In the Netherlands, 7.1% of children grow up in a single-parent household, 0.5% of infants are born to a mother younger than twenty, and 6% of children live in a family that has to rely on welfare [16]. Although several studies indicate a link between low SES and poor health status in early life, there is little insight in the associated healthcare costs. A study in 2019 found lower neighborhood SES to be associated with higher healthcare costs in adults [17]. Two other studies found more frequent healthcare use in young children from families of low SES, and specifically for visits to the general practitioner [18, 19]. However, no studies were found in which both individual and neighborhood-level SES were considered, nor was the information on health status at birth taken into account.

In the Netherlands, basic healthcare insurance is obligatory and uniform across individuals. Children (< 18 years of age) are automatically covered by their parents’ health insurance, without any additional costs. Through health insurance data, insights into healthcare costs can be obtained, preferably with individual level data and with nationwide coverage. Furthermore, the linkage with additional information on non-aggregated SES indicators, enables to assess the relation between SES and healthcare costs on an individual as well as a contextual level. In this study, the healthcare costs in 2014 of all Dutch children between 0 and 3 years and the SES characteristics of monthly household income and neighborhood deprivation index are investigated to examine the relation between SES in early life and healthcare costs. Additionally, since perinatal morbidity is often accompanied by hospital admissions, and subsequent high healthcare costs, but is also more prevalent in low SES situations, the impact of perinatal morbidity on healthcare costs is studied as well.

## Methods

### Data sources

This registry study included data from three different national registries: Perinatal Registration Netherlands for pregnancy outcomes (Perined), Statistics Netherlands for information on socio-economic status (CBS), and Vektis for healthcare costs (Vektis).

Perined contains information on pregnancy, delivery, and neonatal data of more than 97% of all pregnancies in the Netherlands, collected by midwives, gynecologists, and pediatricians [6]. Data about *perinatal morbidity, parity* and *maternal age* at birth from all singleton births from 22 weeks of gestation onwards were obtained from Perined.

The CBS registry added the following information: *monthly household income* (in 2014)*; place of residence* (in 2014)*;* and *ethnicity*. Place of residence was used for linkage with the *deprivation index* (as calculated by Netherlands Institute for Health Services Research (NIVEL)) [20], representing the degree of neighborhood deprivation for the neighborhood in which children grew up.

The Vektis registry holds data on *healthcare costs* in the Netherlands covered by the obligatory basic healthcare insurance. Data were obtained over the year 2014, and linked to all children included in the study cohort.

### Research ethics approval

According to Dutch law, formal ethical assessment of the study protocol was not needed as the study did not involve an intervention and data from CBS are anonymized [based on guidance from the Central Committee on Research Involving Human Subjects (WMO) and the Dutch Personal Data Protection Act]. CBS collects and produces population statistics, referred to as non-public microdata, for all registered Dutch citizens. Under strict conditions, these data are accessible for scientific research. The research board of CBS has reviewed and approved the study protocol (project number 8032). Furthermore, all data and analyses were checked on identifiability of individuals and organizations by an independent employee of CBS before releasing analyses for publication.

### Composition of the study cohort

**Fig. 1:**
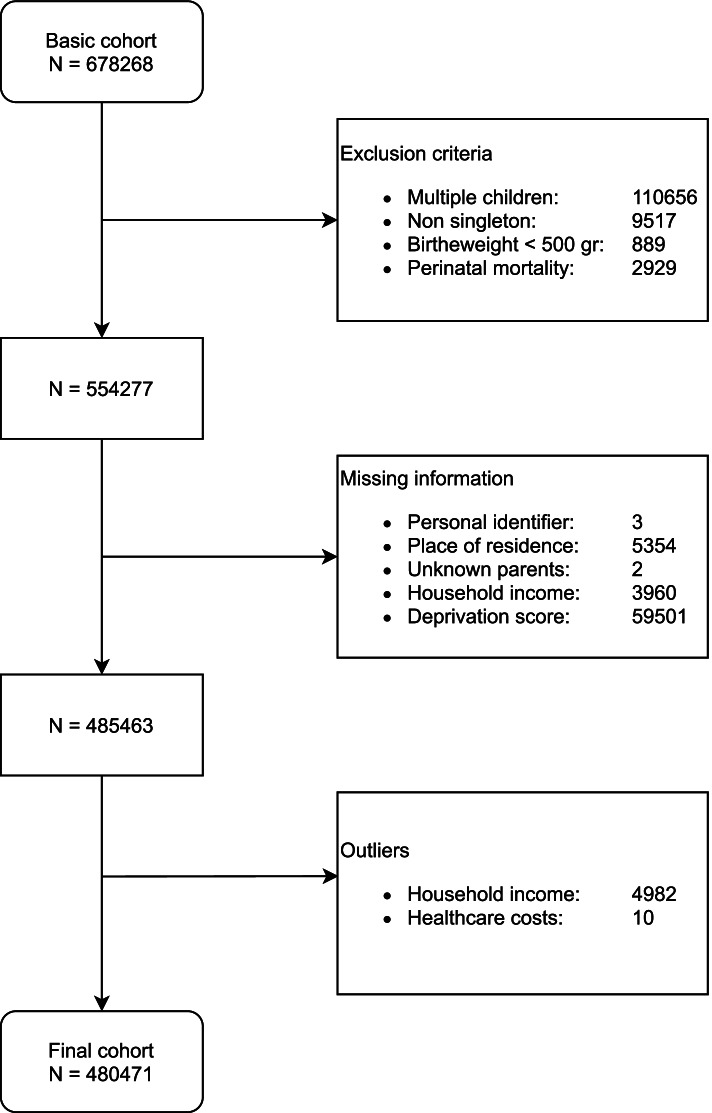
Flowchart of cohort composition and excluded cases

Data from Perined served as the basis of the study cohort and included data on all births between January 1st 2011 and December 31st 2014 (*N* = 678,268). Figure 1 provides a complete overview of the construction of the study dataset. Children were excluded from the study cohort for the following reasons: a) non-singleton children or siblings of an earlier included child in the study cohort (to prevent duplicated information on the family-level); b) non-viability due to low birthweight (< 500 g) or intrauterine fetal death or neonatal death within 1 week postpartum (since these children incurred their healthcare expenses over a very limited period of time); c) missing data on linkage variables (both of child and parent(s)); d) missing information regarding SES (monthly household income and neighborhood deprivation).

### Outcomes

*Healthcare costs:* all healthcare costs covered by basic healthcare insurance spent on the child in 2014. Healthcare is obligatory in the Netherlands, and children under the age of 18 are automatically covered under their parent’s insurance without additional expenses for the parents. It is possible to use additional healthcare services, either paid out of pocket or (partially) covered by additional healthcare insurance, these costs are not included in this study. Healthcare costs are summarized as annual costs and included *primary care costs* (appointments with- and care from the general practitioner and basic mental healthcare); *secondary care costs* (appointments with- and care from medical specialists, nursing on location (at home), hospital stays, surgeries, and emergency treatments); *paramedical care costs* (physiotherapy and therapeutic services such as speech therapy, occupational therapy, and dietary consultations); and *other care costs* (pharmacy, dental care, therapeutic devices, patient transport, healthcare costs made abroad, specialized mental healthcare, and all other costs). All costs are presented in euro’s, and main results are additionally converted to American dollars (USD $) according to the average exchange rate in 2014 (€1 = $1.241), to facilitate international comparison.

### Determinants

#### Main determinants

*Monthly household income*, a continuous determinant representing individual-level SES, was defined as earnings from paid employment, a private company, welfare benefits, retirement pension, and alimony payments. This household income was standardized for household type by CBS to make it comparable across families.

*Neighborhood deprivation,* is captured in the Neighborhood Deprivation Index, which is a continuous determinant representing contextual-level SES. This index was calculated by NIVEL in 2012 based on four CBS neighborhood characteristics: 1) Neighborhood density; 2) Percentage of residents with low income (below 16th income percentile); 3) Percentage of unemployed residents; and 4) Percentage of non-western immigrants [20]. Neighborhood deprivation was only calculated for neighborhoods with > 200 residents, resulting in a relatively high proportion of missing information on this variable (8,8%, see Fig. 1). Furthermore, the formula used to calculate the deprivation index employs individually standardized variables, resulting in an overall neighborhood score of ‘0’ for a neighborhood with all values of the four characteristics similar to the national average of the corresponding characteristics. The index scores range between − 13.26 and 10.7, with higher scores reflecting higher levels of deprivation [20]. These scores were linked to each family through their place of residence, which was defined as the longest residential period within 2014, or in the case of periods of equal length, as the place of residence in the first part of 2014. For descriptive statistics, the index was dichotomized into deprived and non-deprived neighborhoods. The cut-off for this is in line with the recommendations of NIVEL, and places approximately 5% of the total population as inhabitant of a deprived neighborhood (above 4,93) [20].

#### Other determinants

*Perinatal morbidity* was defined as *preterm birth* (< 37 weeks of gestation)*, small for gestational age* ((SGA); birthweight <10th percentile, adjusted for gestational age and fetal sex, according to national reference curves [21])*,* or both. However, the group with both types of perinatal morbidity was small (0·45%), therefore, these children were not included as separate group in the analyses.

*Ethnicity* was defined for all children, based on their parent’s ethnic background, as “native Dutch” or “immigrant”. *Parity*, in which “primiparous” refers to first delivery and “multiparous” to one or more previous deliveries. *Maternal age* is presented continuously in years. *Children’s year of birth* was categorized for each year in the analyses (2011–2014), with children born in 2011 as reference category.

### Data analysis procedure

*Monthly household income, neighborhood deprivation* and *healthcare costs* were checked for outliers. Correction for outliers was performed by removing improbable outliers, resulting in excluding 1% of the data based on *monthly household income* and 0.002% of the data based on *healthcare costs* (excluding negative values and an equal proportion on the highest costs for both variables). *Neighborhood deprivation* showed no outliers. Thus, the final dataset for analyses included data on 480,471 children.

Descriptive analyses were used to summarize the characteristics of the study cohort. Continuous variables were summarized as means (M) with standard deviations (SD) or medians (Mdn) with 5–95% ranges as appropriate. Categorical data were reported as absolute numbers and percentages. Furthermore, per quintile of the main determinants, and categories of the other determinants, healthcare costs were reported. To examine the relation between healthcare costs and SES in early life, binomial logistic regression analyses were performed by building four models in which each consecutive model added to the previous one. For all models, healthcare costs were dichotomized into quartiles with Q1-Q3 in one group (below €1000, reference group) and high healthcare costs (Q4, €1000 or higher).

In model 1, *healthcare costs* were modelled as a function of *preterm birth* and *SGA*. Next, the determinants *maternal age, parity, children’s age,* and *children’s ethnicity* were added in model 2. In model 3, *monthly household income* was added, and in model 4 *neighborhood deprivation* was included. Individual- and contextual-level SES determinants were added in the last models to investigate the effects of these determinants in addition to perinatal health status and background variables.

To assess the generalizability of the findings to a larger population, sensitivity analyses were performed using an extended dataset, also including non-singleton pregnancies and siblings of an earlier included child in the study cohort (*N* = 586,011). Moreover, as robustness checks, both individual- and contextual-level SES determinants were defined in quintiles within the main dataset and *ethnicity* was categorized into three groups (native Dutch, Western immigrant, and non-Western immigrant, according to CBS definitions) [16]. Additional subgroup analyses included the main analyses for each type of *healthcare costs* (*primary care, secondary care, paramedical care*, and *other care*) separately.

Two-sided *p*-values < 0.05 were considered to indicate statistical significance. All analyses were performed in R version 3.4.2 [22].

## Results

Baseline characteristics are presented in Table 1. Approximately 8% of the children in the study cohort resided in deprived neighborhoods, compared to 5% in the nationwide population. Around 13% of children were born with perinatal morbidity (5% born preterm, 8% born SGA). Mothers were on average 30 years old (SD = 4.96), and 54% of children were first born. Overall, children in the study cohort had a median of €295 (€72 to €4299) annual healthcare costs in 2014 (corresponding to a median of $366). In the study cohort, 95% of children had costs in the primary domain; 72% had costs related to other healthcare; 45% in the secondary care domain and 11% had costs related to paramedical care. Healthcare costs of children were generally higher in families living in low SES circumstances, with median costs €73 ($91) higher in the lowest income quintile compared to the highest quintile. Children from families living in the most deprived neighborhoods had €99 ($123) more healthcare costs compared to children living in the most affluent neighborhoods. The highest healthcare costs were found in the first year of the child’s life. Furthermore, compared to their peers without perinatal morbidity, children born preterm had more healthcare costs of €412 ($511), and children born SGA had additional healthcare costs of €66 ($82).

**Table 1:** Distribution of participants and healthcare expenses over determinants, covariates and background variables

		Prevalence ***N*** = 480,471	(%)	Median healthcare costs €	5–95% range €
**Healthcare expenses (per year)^**	··	··	··	295,26	72,22-4299,85
	Primary care	457,221^b^	(95,16%)	13,53 (112,57)^c^	13,53–374,15 (41,08-378,48)^c^
	Secondary care	215,000^b^	(44,75%)	0,00 (726,43)^c^	0,00–3393,35 (64,60-6750,60)^c^
	Paramedical care	53,834^b^	(11,20%)	0,00 (222,76)^c^	0,00–261,13 (40,52-1168,50)^c^
	Other care	346,598^b^	(72,14%)	31,75 (50,33)^c^	0,00–420,73 (13,17-693,54)^c^
**Monthly household income**	Mean household income	1963,99 (1128,38)^a^	··	··	··
	0–20%	··	··	339,56	69,53 – 4925,30
	20–40%	··	··	308,76	73,85 – 4415,00
	40–60%	··	··	290,07	73,62 – 4197,90
	60–80%	··	··	282,37	72,99 – 4059,40
	80–100%	··	··	266,39	71,72 – 3867,00
**Neighborhood deprivation**	Non-deprived	441,748	(91,94%)	289,29	72,01 – 4238,90
	Deprived	38,723	(8,06%)	375,85	78,45 – 4931,50
	0–20%	··	··	257,46	72,20 – 3684,10
	20–40%	··	··	271,91	72,43 – 3968,20
	40–60%	··	··	288,90	71,99 – 4377,70
	60–80%	··	··	312,64	72,00 – 4588,90
	80–100%	··	··	356,36	73,54 – 4926,00
**Perinatal outcome**	Born at term	453,243	(94,33%)	285,74	71,75 – 3548,5
	Born preterm	27,228	(5,67%)	697,49	88,68 – 25,011,70
	Normal birthweight^d^	440,688	(91,72%)	291,37	72,04 – 4059,90
	Small for gestational age	39,707	(8,26%)	357,19	75,54 – 6679,20
	No morbidity	415,691	(86,52%)	281,72	71,50 – 3411,80
	One or both morbidities	64,780	(13,48%)	439,86	80,01–11,885,0
**Ethnicity**	Native Dutch	357,060	(74,31%)	281,79	72,42 – 4239,00
	Western immigrant	38,049	(7,92%)	302,73	67,12 – 3940,38
	Non-western immigrant	85,362	(17,77%)	355,58	75,10– 4689,39
**Children’s year of birth**	2014	94,885	(19,75%)	1562,97	0,00 – 8364,00
	2013	108,121	(22,50%)	297,07	77,60 – 4063,66
	2012	134,528	(28,00%)	215,25	76,01 – 2648,41
	2011	142,937	(29,75%)	230,94	81,57 – 2659,63
**Maternal age**	Mean maternal age	30·35 (4·96)^a^		··	··
**Parity**	Primiparous	258,610	(53,82%)	335,83	72,93 – 5047,15
	Multiparous	221,861	(46,18%)	258,41	71,91–3655,81

^a^Mean and standard deviations are presented, instead of prevalence

^b^Number of children with this type of healthcare cost, children can have multiple types of costs

^c^Median and ranges for children with ≥ €1 of healthcare costs in this category

^d^Missing data: 77 children no birthweight available

There was an unequal distribution of the SES determinants across the other determinants. Children with an immigrant background or born SGA are overrepresented in neighborhoods in the highest deprivation quintile and in families from the lowest income quintile. More specifically, 25.7% of children in our dataset have an immigrant background, with 9.8% of immigrant children living in families with the lowest income quintile, and 10.5% living in the most deprived neighborhoods, as opposed to the expected 5% if these children would be distributed equally over all income and neighborhood deprivation quintiles. Similarly, a total of 8.3% of children in our dataset was born preterm, with 2.3% of children born preterm in families within the lowest income quintile, and 2.1% born in the most deprived neighborhoods, as opposed to the expected 1.7% if these children were distributed equally over all income and neighborhood deprivation quintiles. In contrast, children born preterm seem to be distributed equally across quintiles of both monthly household income and neighborhood deprivation.

As shown in Table 2 children born preterm have an OR of 2.55 (2.48–2.61) compared to children born at term to have high healthcare cost in the first model, and all subsequent models (OR ranging from 2.84 to 2.85). Similarly, SGA children have an OR of 1.41 (1.38–1.45) to 1.44 (1.41–1.48) compared to their peers without SGA to have high healthcare cost in all models. Immigrant children have a slightly higher OR, varying from 1.06 (1.04–1.07) to 1.00 (0.98–1.02) of having high healthcare costs compared to native Dutch children across the models. For every €1000 increase in monthly household income, the OR for having high healthcare costs is 0.99 (0.99–0.99) in both models including this variable. Finally, for every one-unit increase in neighborhood deprivation the OR for having high healthcare costs is 1.02 (1.01–1.02).

**Table 2:** Binomial logistic regression: stepwise model building on the association between healthcare costs and perinatal outcomes, background characteristics, monthly household income and neighborhood deprivation

	Model 1		Model 2		Model 3		Model 4	
	OR	95% CI	OR	95% CI	OR	95% CI	OR	95% CI
Preterm birth	**2,55**	2,48 – 2,61	**2,85**	2,77 – 2,93	**2,84**	2,76 – 2,91	**2,83**	2,76 – 2,91
Small for gestational age	**1,41**	1,38 – 1,45	**1,46**	1,43 – 1,50	**1,45**	1,42 – 1,49	**1,44**	1,41 – 1,48
Maternal age			**0,99**	0,99–0,99	**0,99**	0,99 – 0,99	**0,99**	0,99 – 0,99
Children’s year of birth: 2012			**0,92**	0,90 – 0,94	**0,92**	0,90 – 0,94	**0,92**	0,90 – 0,94
Children’s year of birth: 2013			**1,38**	1,36 – 1,41	**1,38**	1,36 – 1,41	**1,38**	1,35 – 1,41
Children’s year of birth: 2014			**7,70**	7,55 – 7,85	**7,69**	7,54 – 7,84	**7,65**	7,50 – 7,81
Multiparous			0,99	0,98 – 1,02	**0,98**	0,96 – 0,99	**0,98**	0,96 – 0,99
Immigrant status			**1,06**	1,04 – 1,07	**1,03**	1,02 – 1,05	1,00	0,98 – 1,02
Monthly household income					**0,99**	0,99 – 0,99	**0,99**	0,99 – 0,99
Neighborhood deprivation							**1,02**	1,01 – 1,02

**Model 1:** AIC = 528,441

**Model 2:** AIC = 480,333

**Model 3:** AIC = 480,103

**Model 4:** AIC = 479,964

**Healthcare cost – reference group:** €0 - €1001,85

**High healthcare costs:** €1001,85 and higher

**Bold** = *p*-value < 0·05

### Sensitivity and subgroup analyses

Consistent results were obtained in all sensitivity analyses (see Tables 3, 4 and 5).

**Table 3:** Sensitivity analyses with extended sample, including non-singleton pregnancies and siblings of an earlier included child in the study cohort (*N* = 586,011)

	OR	95% CI	***p***
**Preterm birth**	3,14	3,07 – 3,22	< 0,001
**Small for gestational age**	1,48	1,45 – 1,51	< 0,001
**Maternal age**	1,00	0,99 – 1,00	0,58
**Children’s year of birth: 2012**	0,94	0,92 – 0,96	< 0,001
**Children’s year of birth: 2013**	1,48	1,45 – 1,51	< 0,001
**Children’s year of birth: 2014**	7,52	7,38 – 7,66	< 0,001
**Multiparous**	0,88	0,87 – 0,89	< 0,001
**Immigrant**	1,01	0,99 – 1,02	0,36
**Monthly household income**	0,99	0,99 – 0,99	< 0,001
**Neighborhood deprivation**	1,02	1,01 – 1,02	< 0,001

**Table 4:** Sensitivity analyses with monthly household income and neighborhood deprivation categorized into quintiles

	OR	95% CI	***p***
**Preterm birth**	2,83	2,75 – 2,90	< 0,001
**Small for gestational age**	1,44	1,40 – 1,47	< 0,001
**Maternal age**	0,99	0,99 – 0,99	0,034
**Children’s year of birth: 2012**	0,92	0,90 – 0,94	< 0,001
**Children’s year of birth: 2013**	1,38	1,35 – 1,41	< 0,001
**Children’s year of birth: 2014**	7,67	7,52 – 7,82	< 0,001
**Multiparous**	0,96	0,95 – 0,98	< 0,001
**Immigrant**	0,99	0,98 – 1,01	0,440
**Monthly household income Q1**	1,22	1,19 – 1,25	< 0,001
**Monthly household income Q2**	1,22	1,19 – 1,25	< 0,001
**Monthly household income Q3**	1,15	1,12 – 1,17	< 0,001
**Monthly household income Q4**	1,09	1,06 – 1,11	< 0,001
**Neighborhood deprivation Q2**	1,04	1,02 – 1,06	0,001
**Neighborhood deprivation Q3**	1,07	1,05 – 1,10	< 0,001
**Neighborhood deprivation Q4**	1,08	1,05 – 1,10	< 0,001
**Neighborhood deprivation Q5**	1,12	1,10 – 1,15	< 0,001

**Table 5:** Sensitivity analyses with ethnicity categorized into 3 groups (native Dutch background as reference group)

	OR	95% CI	***p***
**Preterm birth**	2,83	2,75 – 2,91	< 0,001
**Small for gestational age**	1,44	1,41 – 1,48	< 0,001
**Maternal age**	0,99	0,99 – 0,99	< 0,001
**Children’s year of birth: 2012**	0,92	0,90 – 0,94	< 0,001
**Children’s year of birth: 2013**	1,38	1,35 – 1,41	< 0,001
**Children’s year of birth: 2014**	7,67	7,52 – 7,83	< 0,001
**Multiparous**	0,97	0,96 – 0,99	< 0,001
**Western immigrant**	0,88	0,86 – 0,91	< 0,001
**Non-western immigrant**	1,07	1,04 – 1,09	< 0,001
**Monthly household income**	0,99	0,99 – 0,99	< 0,001
**Neighborhood deprivation**	1,01	1,01 – 1,02	< 0,001

Subgroup analyses by type of healthcare costs indicated that most children have healthcare costs in the primary care domain, and least in the paramedical care domain (see Table 6). Across all four types of healthcare costs, the effects of monthly household income and neighborhood deprivation index on healthcare costs were similar to the main analyses. The odds for higher healthcare costs related to preterm birth and SGA were higher in the secondary healthcare domain, and lower in all other three domains.

**Table 6:** Subgroup analyses by type of healthcare costs

	Primary care (*N* = 457,221)		Secondary care (*N* = 215,000)		Paramedical care (*N* = 53,834)		Other care (*N* = 346,598)	
	OR	95% CI	OR	95% CI	OR	95% CI	OR	95% CI
**Preterm birth**	**1,26**	0,82 – 0,90	**3,20**	3,10 – 3,31	**1,94**	1,82 – 2,06	**1,99**	1,93 – 2,05
**Small for gestational age**	1,02	0,99 – 1,04	**1,52**	1,47 – 1,57	**1,31**	1,23 – 1,40	**1,14**	1,11 – 1,17
**Maternal age**	**0,97**	0,97 – 0,97	0,99	0,99 – 1,00	**1,01**	1,00 – 1,01	**0,99**	0,99 – 0,99
**Children’s year of birth**: **2012**	**1,32**	1,30 – 1,34	**1,18**	1,14 – 1,22	**0,40**	0,37 – 0,42	**0,85**	0,83 – 0,86
**Children’s year of birth: 2013**	**2,06**	2,02 – 2,10	**1,45**	1,41 – 1,50	**0,38**	0,36 – 0,40	**1,17**	1,15 – 1,20
**Children’s year of birth: 2014**	**0,69**	0,67 – 0,71	**3,36**	3,26 – 3,46	**0,33**	0,31 – 0,35	**0,90**	0,87 – 0,92
**Multiparous**	**0,87**	0,86 – 0,88	**1,09**	1,07 – 1,12	**1,10**	1,05 – 1,15	**1,10**	1,08 – 1,12
**Immigrant**	**1,11**	1,10 – 1,13	**0,94**	0,92 – 0,97	1,03	0,98 – 1,08	**1,17**	1,15 – 1,19
**Monthly household income**	**0,99**	0,99 – 0,99	**0,99**	0,99 – 0,99	**0,99**	0,99 – 0,99	**0,99**	0,99 – 0,99
**Neighborhood deprivation**	**1,03**	1,03 – 1,03	**1,01**	1,01 – 1,02	**1,01**	1,00 – 1,02	**1,02**	1,02 - 1,02

**High primary healthcare costs:** €197.37 and higher

**High secondary healthcare costs:** €1731.53 and higher

**High paramedical healthcare costs:** €438.38 and higher

**High other healthcare costs:** €102.37 and higher

## Discussion

This study investigated the effects of individual- and contextual-level SES adjusted for perinatal morbidity on healthcare costs in early life (0–3 years of age). While controlling for other determinants, for every decrease in household income and increase in neighborhood deprivation, the odds for high healthcare costs increase. Even adjusted for the immediate effects of perinatal morbidity on high healthcare costs, these contextual factors remained of added value.

Analyses revealed differential magnitudes of the association between healthcare costs and children born preterm or SGA. Children born preterm had an OR of 2.55 of higher healthcare cost compared to children born at term, whereas children born SGA had an OR of 1.44 of higher healthcare cost compared to children born with adequate weight. This may be explained by the more multifaceted and heterogeneous origins of premature birth, compared to being born SGA. Risk factors associated with SGA infants focus largely on pregnancy related diseases, maternal characteristics, or medical history [23]. In contrast, preterm birth has a wider variety of underlying causes, such as fetal syndrome (with diverse origins), intrauterine infection or inflammation, or maternal vascular disease [24]. Consequently, premature born children are more severely ill and have an inadequate development, requiring prolonged (and therefore more costly) healthcare.

There are two possible explanations for higher healthcare costs in the low SES population: more ill-health or more (inadequate) healthcare utilization (irrespective of health status). On the one hand, low individual- and contextual-level SES are known to increase the likelihood that individuals will partake in unhealthy, risky, and addictive behaviors, negatively influencing their own health [11–15, 17–19, 25, 26]. In addition, research has shown the effects of low SES to be transgenerational, since children depend on their parents for care and nurturance. The effects of low SES circumstances on (perinatal) health and development are already noticeable in early life [27–33]. Universal health insurance improves access of healthcare services for low SES populations, but does not stop the intergenerational transmission of socioeconomic circumstances and health [28, 34, 35].On the other hand, there is an increased use of (specialized) healthcare services in the lower SES population [13]. Children from families with lower SES might have more need for health services due to higher rates of illness and injury [19]. Conversely, families from low SES may postpone primary care to prevent short term costs, leading to more ill-health and higher costs in the long term [36]. Although healthcare costs of children in the Netherlands are covered by health insurance, adults that postpone their own care are more likely to postpone the care of their children as well, possibly due to an incomplete understanding of the healthcare system.

### Strengths and limitations

This study is based on nearly halve a million children born in the Netherlands, linking data across various routinely collected datasets, and combining both medical and socioeconomic data. Most importantly, due to the large sample size, this study was able to include both individual- and contextual-level measures of SES, while also taking the effects of perinatal morbidity on healthcare costs into account. This duality in the measurement of SES is especially important, given the abundance of scientific evidence on the dissimilarity of individual and contextual SES [30, 37–39]. The difference in estimates between monthly household income and neighborhood deprivation in all models, further demonstrates that these measures should not be assumed to be interchangeable. Moreover, both measures of perinatal morbidity could be included in the model, which are known to be more prevalent in low SES circumstances [31, 40]. Furthermore, the role of individual ethnicity, as well as ethnic density of the neighborhood (as one of the factors contributing to the neighborhood deprivation score) were both considered.

It is important to clearly distinguish between healthcare costs and health status, as healthcare costs cannot be substituted by health status, assuming that higher healthcare costs would indicate poorer health status. Not all healthcare costs are related to ill-health, some are induced by accidents, injuries, or general check-ups, whereas the opposite can also hold true, in which individuals are unhealthy, but do not use healthcare services, and therefore have lower healthcare costs Also, the data represents the expense level of healthcare use, not the underlying conditions causing these healthcare costs. Hence, it is possible that higher healthcare costs are not related to poorer health, but to more frequent or ineffective use of healthcare, irrespective of health-status.

This study also had some limitations. This study used cross-sectional data and thereby ignored the variability of SES circumstances and healthcare costs over time. By applying a cross-sectional analyses strategy, the influence of the variability over time was minimalized. Furthermore, because of the availability of the healthcare cost data per calendar year, it was not possible to calculate healthcare costs per life year of the infant. Additionally, this study lacks information on unhealthiness of the environment of children, such as air pollution, second-hand smoke, or nutrition, both on an individual- and a contextual-level. Individuals of lower SES are more often exposed to these circumstances, which could account for part of the observed associations [10]. Although we were not able to include this in our research, the present study provides a solid base for further exploration of these effects.

### Implication for future research

Our results expand on previous findings linking perinatal morbidity and low SES to higher healthcare costs, by adding the distinction between individual- and contextual-level SES. Nevertheless, this study does not disentangle the underlying mechanism influencing these higher healthcare costs, effects are associations, not causal pathways. Future research should strive to uncover this underlying mechanism further. It could be that the prevalence of ill-health is higher in this population, or that healthcare is used more inefficiently, or a combination of both. Furthermore, it would be of interest to see if healthcare costs of low SES children remain higher across their entire life span, or if this difference converges. And finally, it would be relevant to know if healthcare cost change over time in accordance to changing SES circumstances.

Future research should take into account that healthcare costs in low SES circumstances relates different to preterm birth compared to being born SGA. Therefore, these measures should not be aggregated into one perinatal morbidity factor, but included in analyses as separate variables. Furthermore, this study observed an overrepresentation of children born small for gestational age in low SES circumstances, warranting further investigation on the mechanism behind this. Possibly individual- and contextual-level SES circumstances are associated with maternal stress during pregnancy, hindering optimal fetal growth. However, more research into this hypothesis is necessary.

### Implications for policy

It is important for future research to examine the origins and mechanisms behind differential healthcare costs across SES circumstances. By understanding these mechanisms, policy can be tailored to effectively minimize these differences. In the Netherlands, all children are covered by their parent’s obligatory healthcare insurance without additional costs. Additionally, families with lower household income qualify for healthcare benefits. Consequently, healthcare should be accessible to everyone, irrespective of SES circumstances. Therefore, one of the possible underlying mechanisms could be healthcare use, rather than healthcare access. People of low socioeconomic background have been found to use healthcare services inadequately: they postpone use of primary healthcare services, often resulting in prolonged care and higher costs in the long term. Visits to emergency rooms where primary healthcare would have sufficed, which results in higher immediate healthcare costs are more prevalent in low SES populations [25, 26, 41]. Therefore, by improving health literacy and healthcare access in this population, adequate healthcare use should be facilitated, resulting in lower healthcare costs. By informing parents on health and healthy behaviors, children are exposed to more good examples of healthy behavior at an early age, creating intergenerational positive effects through such policies. Since unhealthy behaviors and unhealthy environments are more prevalent in low SES circumstances, this population should be targeted with high priority.

## Conclusion

To conclude, low SES circumstances are known to influence the health of the child already from pregnancy onwards. Perinatal morbidity was the largest contributor to healthcare costs in young children. On top of this, household income and neighborhood deprivation contributed consistently and independently to higher healthcare costs. Through examination of the underlying mechanisms of differential healthcare costs across SES circumstances, policy can be more effectively tailored to minimize differences in healthcare costs. By optimizing healthcare use of low SES populations the impact of low SES circumstances on their health may be positively influenced. By doing so, not only the health of the mother and child may be optimized, but also future healthcare costs may be reduced.

Additionally, policies that influence more timely and appropriate healthcare use in low SES populations can reduce healthcare costs further.

## Data Availability

Data presented in this study is property of the CBS. Under strict conditions, these data are accessible for scientific research.
